# Computer-Assisted Radiographic Calculation of Spinal Curvature in Brachycephalic “Screw-Tailed” Dog Breeds with Congenital Thoracic Vertebral Malformations: Reliability and Clinical Evaluation

**DOI:** 10.1371/journal.pone.0106957

**Published:** 2014-09-08

**Authors:** Julien Guevar, Jacques Penderis, Kiterie Faller, Carmen Yeamans, Catherine Stalin, Rodrigo Gutierrez-Quintana

**Affiliations:** School of Veterinary Medicine, College of Medical, Veterinary and Life Sciences, University of Glasgow, Glasgow, United Kingdom; University of Sydney, Australia

## Abstract

The objectives of this study were: To investigate computer-assisted digital radiographic measurement of Cobb angles in dogs with congenital thoracic vertebral malformations, to determine its intra- and inter-observer reliability and its association with the presence of neurological deficits. Medical records were reviewed (2009–2013) to identify brachycephalic screw-tailed dog breeds with radiographic studies of the thoracic vertebral column and with at least one vertebral malformation present. Twenty-eight dogs were included in the study. The end vertebrae were defined as the cranial end plate of the vertebra cranial to the malformed vertebra and the caudal end plate of the vertebra caudal to the malformed vertebra. Three observers performed the measurements twice. Intraclass correlation coefficients were used to calculate the intra- and inter-observer reliabilities. The intraclass correlation coefficient was excellent for all intra- and inter-observer measurements using this method. There was a significant difference in the kyphotic Cobb angle between dogs with and without associated neurological deficits. The majority of dogs with neurological deficits had a kyphotic Cobb angle higher than 35°. No significant difference in the scoliotic Cobb angle was observed. We concluded that the computer assisted digital radiographic measurement of the Cobb angle for kyphosis and scoliosis is a valid, reproducible and reliable method to quantify the degree of spinal curvature in brachycephalic screw-tailed dog breeds with congenital thoracic vertebral malformations.

## Introduction

Congenital vertebral malformations causing secondary kyphosis (dorsal curvature of the vertebral column) and scoliosis (lateral curvature of the vertebral column) are relatively common in dogs, especially in the brachycephalic “screw-tailed” breeds such as the English bulldog, French bulldog, Boston terrier and Pug [1]–[3]. Patients with congenital vertebral malformations are often asymptomatic with malformations representing incidental findings identified during unrelated radiographic studies. Clinical signs observed in the affected population are usually those of a progressive myelopathy secondary to vertebral canal stenosis, but also to vertebral instability related to the degree of spinal curvature [1], [2], [4], [5]. The prevalence of clinically affected brachycephalic screw-tailed dogs with congenital vertebral malformations is unknown, but could represent an important “spontaneous” model of spinal deformity.

An important factor evaluated in human patients with congenital vertebral malformations causing kyphosis and scoliosis is the degree of spinal curvature. The angular magnitude of a spinal deformity is usually quantified using the Cobb angle [6]. This method is used to guide decisions regarding progression, physiotherapy, orthotic options and surgical interventions [7]–[9]. Various techniques have been used to determine the Cobb angle in humans, including manual, digital computer-assisted (semi-automatic), automatic and even smartphone procedures [9]–[13].

To the authors' knowledge there are just three previous studies that attempted to quantify the degree of spinal curvature in dogs, and none of them used a computer-assisted method [2], [4], [5]. In the setting of congenital vertebral malformations in dogs, validating a reliable method is an essential first step towards assessment of disease severity, progression, prognostic significance and it may be used to guide treatment. As a first step in understanding the effect of vertebral malformations in dogs, assessing the reliability of a computer-assisted Cobb angle measurement is therefore important.

The aims of the present study were to investigate the use of Cobb angle measurements in dogs with congenital thoracic vertebral malformations in order to objectively quantify the degree of spinal curvature (kyphosis and scoliosis) using a open-access, computer assisted, digital radiographic measurement system and also to determine if the degree of spinal curvature was associated with the presence of neurological deficits in dogs with thoracic vertebral malformations. We hypothesized that the method would be reproducible and reliable and that neurological deficits would be more likely in dogs with more severe kyphosis.

## Materials and Methods

### Ethics statement

This study was considered as sub-threshold for specific ethical approval by the convenor of the school of veterinary medicine ethics committee, as the work involved only analysis of data routinely recorded from normal and necessary clinical procedures.

### Cases

The medical records of the University of Glasgow Small Animal Hospital were retrospectively reviewed from September 2009 to April 2013 to identify French bulldogs, English bulldogs, Boston terriers and Pugs with or without neurological deficits that had lateral and ventro-dorsal digital radiographs of the thoracic spine with at least a single vertebral congenital malformation present. The breed, age and sex were recorded. If there were any neurological deficits associated with the vertebral malformation identified, then the neurological grade at presentation was recorded, using the standard grading system (Table 1) [14]. Patients were then divided into two groups, one where the vertebral malformation was associated with neurological deficits (Group 1) and one without associated neurological deficits (Group 2). The cases used in the present study were also included in a previous study on classification of congenital vertebral malformations [15]. All dogs in group1 had magnetic resonance imaging which confirmed the compressive myelopathy to be secondary to the vertebral malformation on sagittal and transverse T2 weighted images.

**Table 1:** Clinical 0 to 5 grading scale for thoracolumbar spinal cord lesions [14] and incidence in the two groups.

*Grade*	*Clinical signs*	*Group1*	*Group2*
**0**	Normal		16/16 (100%)
**1**	Spinal pain	0/12 (0%)	
**2**	Ambulatory paraparesis	9/12 (75%)	
**3**	Non-ambulatory paraparesis	2/12 (17%)	
**4**	Paraplegia with intact deep pain perception	1/12 (8%)	
**5**	Paraplegia with absent deep pain perception	0/12 (0%)	

### Radiographic assessment

Radiographs of the thoracic spine (lateral and ventro-dorsal views) were performed using a digital radiography system (Siemens, Camberley, United Kingdom). They were then evaluated by three observers using an open-source PACS Workstation DICOM viewer (Osirix Imaging Software, v 3.9.2, Pixmeo, Geneva, Switzerland) that measured the Cobb angle automatically. Observers included two board certified veterinary neurologists (RG, JP) and a veterinary neurology resident (JG) that were blinded to the dog groups. Radiographs were reviewed on the same monitor of a laptop computer (Mac Book Pro, Apple, Cupertino, California, USA) on two occasions and several weeks apart.

**Figure 1:**
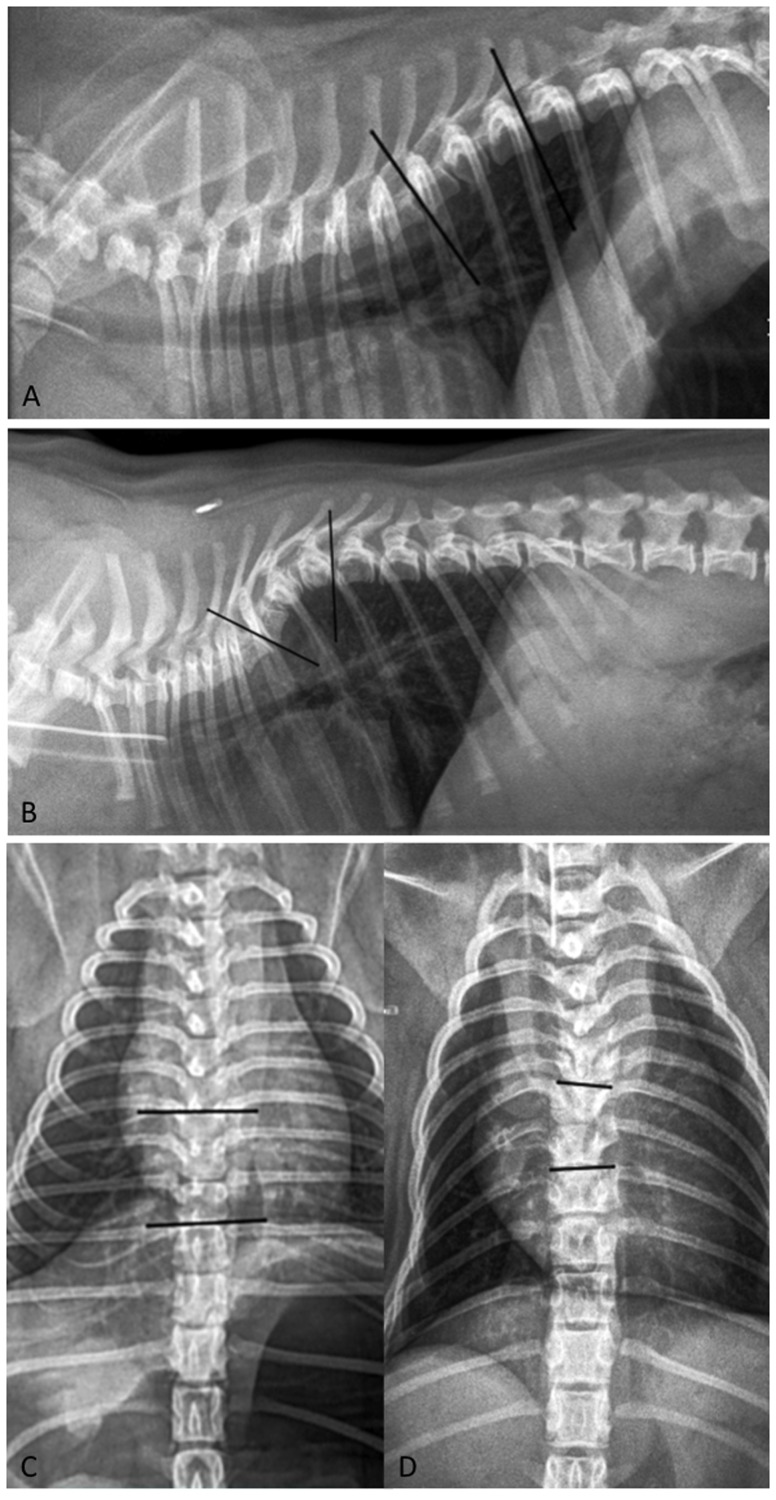
Cobb angles measurements. Lateral and ventro-dorsal radiographs of a neurologically normal Pug with a congenital vertebral malformation at the eighth thoracic vertebra (T8) identified as an incidental finding (A,C) and a clinically affected Pug with a related congenital vertebral malformation at the seventh thoracic vertebra (T7) (B,D). The placement of the reference lines for the calculation of the kyphotic (lateral radiograph) and scoliotic (ventro-dorsal radiograph) angles is shown. The lines pass over the cranial vertebral end plate of T7 and over the caudal vertebral end plate of the ninth thoracic vertebra (T9) in the normal pug (A,C); and similarly over the vertebral end plates of the sixth thoracic vertebra (T6) and T8 in the affected pug (B,D).

The degree of spinal curvature was assessed on the ventro-dorsal view for scoliosis and on the lateral view for kyphosis of the vertebral malformation leading to spinal curvature. Two reference lines, including a line parallel to the cranial vertebral end plate of the first vertebra cranial to the malformed vertebra and a line parallel to the caudal vertebral end plate of the first caudal vertebra, were traced and the Cobb angle was automatically calculated by the software (Figure 1). If multiple malformations were present, each was evaluated individually unless they were adjacent, in which case the most significant vertebral malformation was selected.

### Statistics

The intraclass correlation coefficient (ICC) two-way mixed model on absolute agreement was used to analyse the measurement reliability and it was calculated for both intra- and inter-observer reliability [16]. The value can range from zero to one, with a higher value indicating better reliability. ICC less than 0.40 was considered as poor; 0.40 to 0.59 as fair; 0.60 to 0.74 as good, and 0.75 to 1.00 as excellent [17]. Descriptive statistics were reported as mean, median, range and standard deviation (SD). The Mann-Whitney test was used to compare both groups as the data was not normally distributed. Statistical significance was set for *P*<0.05. When statistics were calculated to compare the two groups, only one set of values from one observer was used (as the ICC values were excellent). When multiple vertebral malformations were present the one with the highest kyphotic and or scoliotic angle was used for the comparison between groups. Data was analyzed using statistical software (Minitab 16.0, Minitab Inc, Coventry, UK and SPSS 21, IBM Corp, Chicago, IL, USA).

## Results

Data on the groups and the neurological status are summarized in Table 1 and Table 2. Twenty-one dogs had a single congenital vertebral malformation causing some degree of kyphosis and/or scoliosis, five had two malformations and two had three malformations.

**Table 2:** Incidence, breed, sex and age (n = 28).

*Variables*	*Group1*	*Group2*	*Total population*
**Population**	12 (43%)	16 (57%)	28 (100%)
**Breed**	Pug: 9 (75%)	Pug: 2 (12.5%)	Pug: 11 (39%)
	EB: 2 (17%)	EB: 9 (56%)	EB: 11 (39%)
	BT: 0	BT: 3 (19%)	BT: 3 (10.5%)
	FB: 1 (8%)	FB: 2 (12.5%)	FB: 3 (10.5%)
**Sex**	Female: 6 (50%)	Female: 2 (12.5%)	Female: 8 (28%)
	Male: 6 (50%)	Male: 14 (87.5%)	Male: 20 (72%)
**Age**	Mean: 2.4 y	Mean: 2.6 y	Mean: 2.5 y
	Range: 4 m-7.5 y	Range: 7 m-14 y	Range: 4 m-14 y
	SD: 2.4 y	SD: 3.2 y	SD: 2.86 y

EB: English bulldog, FB: French bulldog, BT: Boston terrier, m: months, y: years and SD: Standard deviation.

The mean Cobb angle of all measured radiographs for kyphosis was 24.02° (n: 222; median: 16.94°; range: 0.02°–87°), and 5.69° for scoliosis (n: 222; median: 3.02°; range: 0°–49.72°). The mean Cobb angle for kyphosis of group 1 (dogs with associated neurological deficits) was 45.67° (n: 72; median: 49.35°, range: 7.7°–87°) and 16.89° for group 2 (dogs without associated neurological deficits) (n: 96; median: 14.62°; range: 1.50°–47.90°). The mean Cobb angle for scoliosis of group 1 was 9.35° (n: 72; median: 4.10°; range 0°–49.72°) and 3.82° (n: 96; median: 2.39°; range: 0°–17.6°) for group 2.

**Figure 2:**
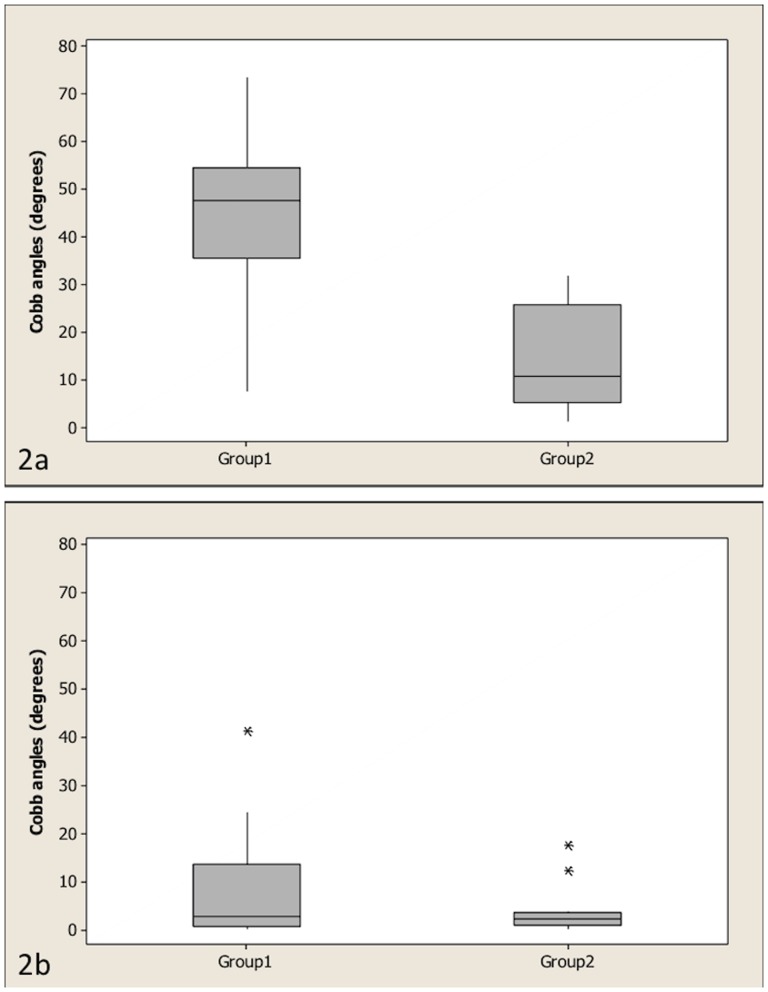
Boxplots of the two groups. Boxplots of the kyphotic Cobb angles for group1 (dogs with associated neurological deficits) and group 2 (dogs without associated neurological deficits) (2a) (*P*<0.001) and boxplots of the scoliotic angles of groups 1 and 2 (2b) (*P* = 0.55). The Mann-Whitney test was used to compare both groups. The bottom and top lines of the box represent the first and third quartiles, the band inside the box represents the median and the asterisks outside the box and whisker plot represent outliers.

There was a statistically significant difference between the kyphotic angles of the two groups (*P*<0.001), but there was no statistically significant difference between its scoliotic angles (*P* = 0.55) (Figure 2). A kyphotic angle >35° had a positive predictive value of 75% for related neurological deficits (negative predictive value of 100%), with a sensitivity and specificity of 100% and 84% respectively.

The intra-and the inter-observer ICC were both excellent when assessing the Cobb angle to quantify spinal curvature on digital radiographs; when the confidence interval was set at 95% (CI 95%), it remained excellent. Table 3 outlines the intra- and inter-observer correlation coefficients for the Cobb angles and their CI 95%.

**Table 3:** Intraclass correlation coefficient (ICC) and 95% coefficient intervals for digital measurement of the Cobb angles (n = 222).

		*Data*	*ICC*	*CI 95%*
		Intraobserver1	0.996	0.992–0.998
		Intraobserver2	0.989	0.980–0.995
	Kyphosis	Intraobserver3	0.984	0.969–0.992
		Interobserver1,2,3	0.982	0.967–0.990
		Interobserver,2,3	0.972	0.950–0.985
**Cobb angles**				
		Intraobserver1	0.99	0.980–0.995
		Intraobserver2	0.958	0.915–0.979
	Scoliosis	Intraobserver3	0.936	0.876–0.967
		Interobserver1,2,3	0.957	0.926–0.976
		Interobserver1,2,3	0.964	0.936–0.981

CI: Coefficient Interval; 1,2,3: refer to the 3 observers. ICC: intra class correlation coefficients.

## Discussion

To the authors' knowledge, this is the first study assessing the reliability of a computer-assisted digital radiographic measurement method to calculate the Cobb angle in veterinary medicine, despite the method being one of the most commonly used in human medicine for assessment of kyphosis and scoliosis [18], [19]. We confirmed the hypothesis that the evaluation of the Cobb angle on digital radiographs is a feasible and reliable method to quantify the degree of spinal curvature in dogs. Three previous veterinary studies investigated spinal curvature in dogs: one studied a manual technique to measure the degree of kyphosis and its relationship with neurological deficit, and the other two mentioned the use of the Cobb angle manual technique to assess the deformity prior to surgery in dogs without assessing its reliability [2], [4], [5].

As digital imaging techniques are available in most veterinary practices and hospitals, we aimed to evaluate the feasibility and the reproducibility of Cobb angle measurements in patients with abnormal spinal curvatures, such as kyphosis and scoliosis, via a computer assisted, commercially available plug-in for digital radiography. We confirmed that this method was easy to use and that its reproducibility was excellent, with ICC values over 0.9. When assessing the confidence interval set at 95%, we could conclude that it remained excellent. Assessing this method on digitalized radiography offered the advantage of evaluating a technique which is accessible for general practitioners, and where image size and contrast could be modulated to better define the vertebral end plates, without influencing the results.

Cobb angle measurements have been reported to have a high variability due to incorrect definition of the end vertebra, as well as defective angle measurement [20]. The rationale for the use of a software plug-in that automatically calculates the Cobb angle and for the use of pre-selected and constant end-vertebrae was to reduce these sources of error [20], [21]. It is clear however that inaccuracy in angle measurement exists and was inherent to the method itself as measurement of a three-dimensional structure was attempted in a two-dimensional radiographic plane. This was in part explained by the difficulty in drawing a line through a vertebral end plate, which was not evident as a straight line on the radiographs, as well as the difficulty in defining some of the vertebral endplates when vertebrae were superimposed, in particular on ventro-dorsal view radiographs.

The mean scoliotic angles for the two groups were lower than 10°, which should therefore not be classified as scoliosis *per se* [22]. This, in part, explains why scoliosis was identified in many dogs in our population, without it being related to the presence of neurological deficits.

Kyphosis was the main spinal deformity observed in association with clinical signs, as previously reported [1], [2], [5]. In the affected group of 12 dogs, in two dogs the neurological deficits could be ascribed to extrusion of an intervertebral disc adjacent to the congenital vertebral malformation or due to empyema secondary to discospondylitis adjacent to the congenital vertebral malformation. Early degeneration of intervertebral discs adjacent to malformed vertebrae has recently been highlighted in dogs, and may have been the predisposing factor in that patient [23]. If those two cases are excluded from our affected population, then a cut-off appears around a kyphotic angle of 35°. This finding would suggest that, if indeed kyphotic angles are of progressive nature, [1], [24] healthy young dogs with a kyphotic angle below, but close to, 35° may benefit from monitoring for the subsequent appearance of neurological deficits. Serial Cobb angle measurements in these patients would also allow objective determination of progression of vertebral angulation. There is currently no supportive data to suggest any advantages for preventive stabilization with spinal surgery in dogs without neurological deficits; although early intervention may be warranted to prevent them.

Although this study has limitations in its retrospective nature and its small sample size, it advances evidence for the computer-assisted digital radiographic measurement of the Cobb angle as a reliable, reproducible and easy to use method to quantify the degree of spinal curvature in dogs. Further prospective studies, most likely multicenter with larger populations, would be valuable to assess the exact correlation between the degree of spinal curvature and the neurological status in pre-defined dog breeds; as determined by the Cobb angle measurement.

## Conclusions

The goal of this study was to assess the use of the Cobb angle method in dogs with spinal curvature and to determine the interobserver and intraobserver reliability of the technique. We concluded that the use of digital imaging and software allowed accurate and reproducible measurement of the Cobb angle in a canine group with congenital vertebral malformations. We also identified in our groups, that a Cobb angle value greater than 35° indicated a high probability of related neurological deficits.

## Supporting Information

Data S1 **The data used in the present study is available as a supplementary file.**
(XLSX)

## References

[1] Westworth DR, Sturges BK (2010) Congenital spinal malformations in small animals. Vet Clin North Am Small Anim Pract 40: 951–981. 20732600 10.1016/j.cvsm.2010.05.009

[2] Moissonier P, Gossot P, Scotti S (2011) Thoracic kyphosis associated with hemivertebra. Vet Surg 40: 1029–1032. 22091966 10.1111/j.1532-950X.2011.00876.x

[3] Done SH, Drew RA, Robins G, Robins GM, Lane JG (1975) Hemivertebra in the dog: clinical and pathological observations. Vet Record 96: 313–317. 10.1136/vr.96.14.313 1129930

[4] Aikawa T, Shibata M, Asano M, Hara Y, Tagawa M, et al (2014) A Comparison of Thoracolumbar Intervertebral Disc Extrusion in French Bulldogs and Dachshunds and Association With Congenital Vertebral Anomalies. Vet Surg 43: 301–307. 24433331 10.1111/j.1532-950X.2014.12102.x

[5] Aikawa T, Kanazono S, Yoshigae Y, Sharp NJ, Muñana KR (2007) Vertebral Stabilization Using Positively Threaded Profile Pins and Polymethylmethacrylate, with or Without Laminectomy, for Spinal Canal Stenosis and Vertebral Instability Caused by Congenital Thoracic Vertebral Anomalies. Vet Surg 36: 432–441. 17614924 10.1111/j.1532-950X.2007.00289.x

[6] Cobb J (1948) Outline for the study of scoliosis. Am Acad Orthop Surg Instr Course Lect 5: 261–275.

[7] Nasca RJ, Stelling FH, Steel HH (1975) Progression of congenital scoliosis due to hemivertebrae and hemivertebrae with bars. J Bone Joint Surg Am 57: 456–466. 1141254

[8] McMaster M, Singh H (1999) Natural history of congenital kyphosis and kyphoscoliosis: A study of one hundred and twelve patients. J Bone Joint Surg Am 81: 1367–1383. 10535587 10.2106/00004623-199910000-00002

[9] Langensiepen S, Semler O, Sobottke R, Fricke O, Franklin J, et al (2013) Measuring procedures to determine the Cobb angle in idiopathic scoliosis: a systematic review. Eur Spine J 22: 2360–2371. 23443679 10.1007/s00586-013-2693-9 PMC3886494

[10] Srinivasalu S, Modi H, Smehta S, Suh SW, Chen T (2008) Measurement of scoliosis and kyphosis radiographs. Intraobserver and interobserver variation. Asian Spine J 2: 90–93. 20404962 10.4184/asj.2008.2.2.90 PMC2852087

[11] Tanure MC, Pinheiro AP, Oliveira AS (2010) Reliability assessment of Cobb angle measurements using manual and digital methods. Spine J 10: 769–774. 20359959 10.1016/j.spinee.2010.02.020

[12] Zhang J, Lou E, Hill D, Raso JV, Wang Y, et al (2010) Computer-aided assessment of scoliosis on posteroanterior radiographs. Med Biol Eng Comput 48: 185–195. 20012376 10.1007/s11517-009-0556-7

[13] Qiao J, Liu Z, Xu L, Wu T, Zheng X, et al (2012) Reliability analysis of a smartphone-aided measurement method for Cobb angle scoliosis. J Spinal Disord Tech 25: E88–E92. 22237178 10.1097/BSD.0b013e3182463964

[14] Sharp N, Wheeler S (2005) Thoracolumbar disc disease. In: Small animal spinal disorders: Diagnosis and surgery Sharp N, Wheeler S, Editors. Philadelphia, Elsevier Mosby. pp. 121–159.

[15] Gutierrez-Quintana R, Guevar J, Stalin C, Faller K, Yeamans C, et al.. (2014) A proposed radiographic classification of congenital thoracic vertebral malformations in brachycephalic “screw-tailed” dog breeds. Vet Radiol Ultrasound In Press. 10.1111/vru.12172 24833506

[16] Shrout P, Fleiss J (1979) Intraclass correlations: Uses in assessing rater reliability. Psychol Bull 86: 420–428. 18839484 10.1037//0033-2909.86.2.420

[17] Fleiss JL (1986) Reliability of Measurement. In: The Design and Analysis of Clinical Experiments Fleiss JL, editor. Toronto, Wiley. pp1–32.

[18] Harrison DE, Harrison DD, Cailliet R, Janik TJ, Holland B (2001) Radiographic analysis of lumbar lordosis: centroid, cobb, TRALL, and Harrison posterior tangent methods. Spine 26: E235–E242. 11389407 10.1097/00007632-200106010-00003

[19] Morissy RT, Goldsmith GS, Hall EC, Kehl D, Cowie GH (1990) Measurement of the Cobb Angle on radiographs of patients who have scoliosis. J Bone Joint Surg Am 72: 320–327. 2312527

[20] Gstoetner M, Sekyra K, Wallochnik N, Winter P, Wachter R, et al (2007) Inter-and intraobserver reliability assessment of the Cobb angle: manual versus digital measurement tools. Eur Spine J 16: 1587–1592. 17549526 10.1007/s00586-007-0401-3 PMC2078306

[21] Shea KG, Stevens PM, Nelson M, Smith JT, Masters KS, et al (1998) A comparison of manual versus computer-assisted radiographic measurement. Intraobserver measurement variability for Cobb angles. Spine 23: 551–555. 9530786 10.1097/00007632-199803010-00007

[22] Angevine PD, Deutsch H (2008) Idiopathic scoliosis. Neurosurgery 63: A86–A93. 10.1227/01.NEU.0000320427.28377.F7 18812937

[23] Faller K, Penderis J, Stalin C, Guevar J, Yeamans C, et al (2014) The effect of kyphoscoliosis on intervertebral disc degeneration in dogs. Vet J 200: 449–451. 24745767 10.1016/j.tvjl.2014.03.027

[24] Philips MF, Dormans J, Drummond D, Schut L, Sutton LN (1997) Progressive Congenital Kyphosis: Report of Five Cases and Review of the Literature. Pediatr Neurosurg 26: 130–143. 9419030 10.1159/000121178

